# Zoledronic acid induces antiproliferative and apoptotic effects in human pancreatic cancer cells *in vitro*

**DOI:** 10.1038/sj.bjc.6600986

**Published:** 2003-06-10

**Authors:** P Tassone, P Tagliaferri, C Viscomi, C Palmieri, M Caraglia, A D'Alessandro, E Galea, A Goel, A Abbruzzese, C R Boland, S Venuta

**Affiliations:** 1 Oncology Unit, Department of Experimental and Clinical Medicine, Via T. Campanella, 115 ‘Magna Græcia’ University, 88100 Catanzaro, Italy; 2 Department of Medicine and Comprehensive Cancer Center, University of California, San Diego, La Jolla, CA, USA; 3 Department of Biochemistry and Biophysics, II University of Naples, Italy

**Keywords:** pancreatic cancer, zoledronic acid, zoledronate, zometa, bisphosphonates, caspase-9, p21^ras^, apoptosis

## Abstract

Bisphosphonates (BPs) are an emerging class of drugs mostly used in the palliative care of cancer patients. We investigated the *in vitro* activity of the most potent antiresorptive BP, zoledronic acid (ZOL), on the growth and survival of three human pancreatic cancer (PC) cell lines (BxPC-3, CFPAC-1 and PANC-1). Pancreatic cancer frequently has a dysregulated p21^ras^ pathway and therefore appears to be a suitable target for BPs that interfere with the prenylation of small GTP-binding proteins such as p21^ras^. We found that ZOL induces growth inhibition (IC_50_:10–50 *μ*M) and apoptotic death of PC cells. The proapoptotic effect was correlated to cleavage/activation of caspase-9 and poly(ADP)-ribose polymerase, but not of caspase-3. Moreover, we studied the p21^ras^ signalling in cells exposed to ZOL and detected a reduction of p21^ras^ and Raf-1 content and functional downregulation of the terminal enzyme ERK/MAPkinase and of the pKB/Akt survival pathway. Finally, we observed that ZOL induces significant cytoskeletal rearrangements. In conclusion, we demonstrated that ZOL induces growth inhibition and apoptosis on PC cells and interferes with growth and survival pathways downstream to p21^ras^. These findings might be relevant for expanding application of BPs in cancer treatment.

Cancer of the exocrine pancreas is the fourth leading cause of cancer-related deaths in both men and women (Fernandez *et al*, 1994). Even if advances in surgical techniques, radiation and chemotherapy have provided significant improvements in the overall survival and on the quality of life, fewer than 5% of pancreatic cancer (PC) patients survive 5 years after diagnosis (Parker *et al*, 1996). Therefore, it is not surprising that PC is a serious health problem. Thus, there is a great demand for new and more effective therapeutic strategies for the treatment of this disease. Recent studies on pancreatic tumour tissues and cell lines have shown that multiple subsets of genes undergo activation or inactivation during development and progression of disease (Hilgers and Kern, 1999). Specific point mutations at codon 12 of the K-*ras* oncogene are detected in 75–90% of PC specimens and constitute the most common genetic changes in PC (Almoguera *et al*, 1988). The mutated K-*ras* protein is not able to convert GTP to GDP, resulting in constitutively active GTP-bound species and constitutive activation of cell proliferative signals. It is therefore conceivable that the pancreatic carcinogenetic process and/or the maintenance of the transformed phenotype strongly rely on the dysregulated activity of the p21^ras^-mediated signalling. The inhibition of p21^ras^ signalling has therefore been postulated as a possible target for anticancer therapy and might be specifically suitable for the treatment of PC.

Bisphosphonates (BPs), the most effective and widely used class of drugs in the treatment of metabolic bone disorders, including tumour-associated bone disease, are capable of inhibiting p21^ras^ signalling (Luckman *et al*, 1998; Green, 2001). Therefore, the extensive evaluation of these compounds, and particularly the study of the recently available and more potent nitrogen-containing BPs, may suggest expanding applications of these drugs and warrant further investigation. It is well known that BPs are compounds that strongly bind to hydroxyapatite crystals and accumulate in mineralised bone (Jung *et al*, 1973). Bisphosphonates, which are specifically internalised into osteoclasts (Sato *et al*, 1991), lead to inhibition of cell function because of changes in the cytoskeleton and loss of the ruffled border (Carano *et al*, 1990), as well as apoptosis (Hughes *et al*, 1995). Third-generation, nitrogen-containing BPs, such as zoledronic acid (ZOL) (Green *et al*, 1994), inhibit farnesyldiphosphate (FPP) synthase, an enzyme involved in the mevalonate pathway (Luckman *et al*, 1998; Benford *et al*, 1999), preventing post-translational events of prenylation of small GTP-binding proteins such as p21^ras^, Rab, Rho, Rac and cdc42 (Luckman *et al*, 1998), which are required for a variety of biological functions including signal transduction and cell adhesion.

In addition to the potent antiresorptive effects of nitrogen-containing BPs, recent reports have also shown that these compounds induce antiproliferative and apoptotic effects in multiple myeloma cells *in vitro* (Shipman *et al*, 1997; Aparicio *et al*, 1998), may synergise with chemotherapeutic or biological agents (Tassone *et al*, 2000, 2002), and may offer clinical benefits (Dhodapkar *et al*, 1998; Berenson *et al*, 1998). A direct effect induced by BPs has also been demonstrated on tumour cells of nonhaematopoietic origin. Bisphosphonates induce inhibition of adhesion of breast and prostate cancer cells to bone matrix (Van Der *et al*, 1996; Boissier *et al*, 1997). In a mouse model of breast cancer, BPs inhibited the progression and the development of bone metastasis (Sasaki *et al*, 1995). Other studies have also suggested that BPs may interfere with the growth and survival of metastatic cancer cells in bone (Pelger *et al*, 1998). More recently, a variety of studies have focused on the direct effect of BPs on growth and survival of cancer cells from solid tumours. ZOL induces a significant reduction of cell viability, increases apoptotic cell death and downregulation of bcl-2 protein, p21^ras^ delocalisation from the cell membrane and proteolytic cleavage of PARP, indicating direct antitumour effects on human breast cancer cells (Senaratne *et al*, 2000; Senaratne *et al*, 2002). In prostate cancer cells, ZOL has shown a remarkable inhibitory effect on cell proliferation by induction of cell death and/or cytostasis *in vitro* (Lee *et al*, 2001).

In this scenario, since there have been no studies addressing the possibility of a direct effect of BPs on growth and survival of human PC cells, we studied the activity of the most potent BP, ZOL, on a panel of different human PC cell lines (BxPC-3, CFPAC-1 and PANC-1). We have additionally studied whether the nitrogen-containing ZOL might induce effects on the p21^ras^/raf1/MEK1/ERK and on the pkB/Akt pathways, and if apoptosis should be related to caspase-9/-3 and PARP cleavage/activation in these cancer cells.

## MATERIALS AND METHODS

### Cell cultures and reagents

The PC cell lines BxPC-3, CFPAC-1 and PANC-1 were purchased from the American Type Culture Collection (Rockville, MD, USA). BxPC-3 and CFPAC-1 are adherent human pancreatic adenocarcinoma cell lines that were grown in RPMI 1640 and Iscove's modified Dulbecco's media, respectively. PANC-1 is a human epithelioid pancreas carcinoma cell line that was grown in Dulbecco's modified Eagle's medium (DMEM). All media were supplemented with 10% heat-inactivated fetal bovine serum, 2 mM L-glutamine, 100 *μ*g ml^−1^ streptomycin and 100 U ml^−1^ penicillin. All PC cell lines were cultured at a constant temperature of 37°C in a humidified atmosphere of 5% carbon dioxide (CO_2_). The bisphosphonic acid monohydrate ZOL, 1-hydroxy-2-[(1H-imidazol-1-yl) ethylidene], a third-generation BP, was kindly provided as the hydrated disodium salt by Novartis Pharma AG (Basel, Switzerland). The neutralised sodium salt of ZOL was dissolved in sterile ddH_2_O and used at a final concentration in a range of 1–100 *μ*M. Stock solutions of ZOL were aliquoted and kept at −20°C for long-term storage. Anti-caspase-9/-3 and anti-PARP MAbs were purchased from New England Biolabs (Beverly, CA, USA). Protein sepharose was purchased from Sigma (St Louis, MO, USA). Rabbit antisera raised against raf-1 C-12, *β*actin, ERK-1 K-23 and ERK-2 MAb C-14 were purchased from Santa Cruz Biotechnology (Santa Cruz, CA, USA). Anti-pan-ras MAb clone 10 was purchased from Calbiochem (San Diego, CA, USA). Anti-Akt MAbs, GSK3*β* fusion protein and anti-pGSK3*β* MAbs were all from Cell Signaling Tech. (Beverly, MA, USA).

### Cell proliferation assay

Analysis of cell proliferation was performed on PC cells in the presence of increasing concentrations of ZOL by the MTT assay. Briefly, PC cells (3 × 10^4^ well^−1^) were seeded in 96-well plates in serum-containing media and allowed to attach for 24 h. The medium was then removed and replaced with new medium containing ZOL at different concentrations. Cells were incubated under these conditions for a time course spanning 72 h. Then cells were incubated with 10 *μ*l well^−1^ of thiazolyl blue (MTT, 5 mg ml^−1^, Sigma) for 1 h at 37°C. After incubation, 100 *μ*l of 0.04 N HCl in isopropanol was added into each well and the absorbance was measured at a wavelength of 620 nm in a microplate reader. A value of 100% was assigned to untreated control cultures, and the IC_50_ was defined as the concentration of drug that reduced the number of viable cells to 50% of the control cultures after 72 h of exposure.

### Flow cytometric analysis of apoptosis

Apoptotic cell death was analysed by Annexin-V–FITC staining and by propidium iodide (PI) detection systems. Annexin-V–FITC binds to phosphatidylserine residues, which are translocated from the inner to the outer leaflet of the plasma membrane during the early stages of apoptosis. Labelling of apoptotic cells was performed using an Annexin-V kit (MedSystems Diagnostics, Vienna, Austria). Briefly, cells were incubated with Annexin-V–FITC in a binding buffer (provided by the manufacturer) for 10 min at room temperature, washed and resuspended in the same buffer as described by the manufacturer. Analysis of apoptotic cells was performed by flow cytometry (FACScan, Becton Dickinson). Propidium iodide analysis of apoptosis was performed using a commercial kit (MedSystems Diagnostics, Vienna, Austria). The cells were washed in PBS, resuspended in 190 *μ*l of prediluted binding buffer (1 : 4) and incubated for 10 min with 10 *μ*l of the 20 *μ*g ml^−1^ propidium iodide stock solution, and then the apoptotic cells were analysed by FACScan flow cytometer. For each sample, 2 × 10^4^ events were acquired. Analysis was carried out by triplicate determination on at least three separate experiments. Induction of apoptotic cell death was further performed in the presence or absence of specific caspase inhibitors: VAD-fmk(-fluoromethylketone) which is a caspase-9 inhibitor, VEID-fmk which is specific for caspase-6 and DEVD-fmk which is a caspase-3 inhibitor.

### Immunoblotting for p21^ras^, raf-1, ERK, pERK, caspase-9/-3, Akt

For extract preparation, the cells were washed twice with ice-cold PBS/BSA, scraped and centrifuged for 30 min at 4°C in 1 ml of lysis buffer (1% Triton, 0.5% sodium deoxycholate, 0.1N NaCl, 1 mM EDTA, pH 7.5, 10 mM Na_2_HPO_4_, pH 7.4, 10 mM phenylmethylsulphonyl fluoride (PMSF), 25 mM benzamidin, 1 mM leupeptin, 0.025 U ml^−1^ aprotinin). Equal amounts of cell proteins were separated by SDS–PAGE. The proteins on the gels were electrotransferred to nitrocellulose and reacted with the different MAbs. Nitrocellulose filters were subsequently exposed to HRP-conjugated secondary antibodies. Finally, the film was washed with PBS/0.05% Tween 20 and immunoreactive proteins were detected by the ECL, chemiluminescence technique (Amersham, Uppsala, Sweden).

### Analysis of pKB/Akt activity

Akt assay was performed using reagents supplied as a kit obtained from Cell Signaling Technology Inc. (Cell Signaling Tech., Beverly, MA, USA). Briefly, 10^6^ PC cells were lysed in a buffer containig 20 mM Tris (pH 7.5), 150 mM NaCl, 1 mM EDTA, 1 mM EGTA, 1% Triton X-100, 2.5 mM sodium PP_i_, 1 mM *β*-glycerophosphate, 1 mM Na_3_VO_4_ and 1 *μ*g ml^−1^ leupeptin. Equal amounts of lysed proteins were immunoprecipitated overnight with sepharose-coupled anti-Akt antibodies at 4°C. Immunocomplexes were washed four times with lysis buffer and twice with kinase buffer composed of 25 mM Tris (pH 7.5), 5 mM *β*-glycerophosphate, 2 mM DTT, 0.1 mM Na_3_VO_4_ and 10 mM MgCl_2._ Immune complexes were resuspended in 40 *μ*l of kinase buffer, supplemented with 200 *μ*M ATP and 1 *μ*g of GSK-3*α*/*β* fusion protein, and incubated at 30°C for 30 min. The kinase reaction was stopped by the addition of 20 *μ*l of 3 × SDS sample buffer. Samples (20 *μ*l) were analysed by Western blotting with antiphospho-GSK-3*α*/*β* antibody after PAGE separation.

### Immunofluorescence detection of actin

Pancreatic cancer cells were seeded onto 35-mm culture dishes on sterile coverslips and allowed to attach for 24 h. Subsequently, the cells were incubated for 48 h in the presence of ZOL (15 *μ*M). The medium was then removed, cells washed with PBS and fixed with 1 ml Cytofix/cytoperm (Pharmigen, CA, USA) for 45 min at 4°C. Subsequently, the fixation buffer was removed and cells washed with 1 ml of 1 × washing buffer. For visualisation of filamentous actin, the cells were exposed to rhodamine–phalloidin (100 *μ*g ml^−1^) (Sigma) for 10 min at 4°C and washed with washing buffer. After final washes, coverslips were mounted on the dishes using a 50% solution of glycerol in PBS. The cells were examined under a LEICA TCS SP2 confocal microscope.

### Statistical analysis

Results are expressed as mean ±s.e. The statistical significance of differences between the experimental points was analysed using the *t*-test; differences were considered significant when *P*<0.05.

## RESULTS

### Zoledronic acid induces antiproliferative effects on PC cells

**Figure 1:**
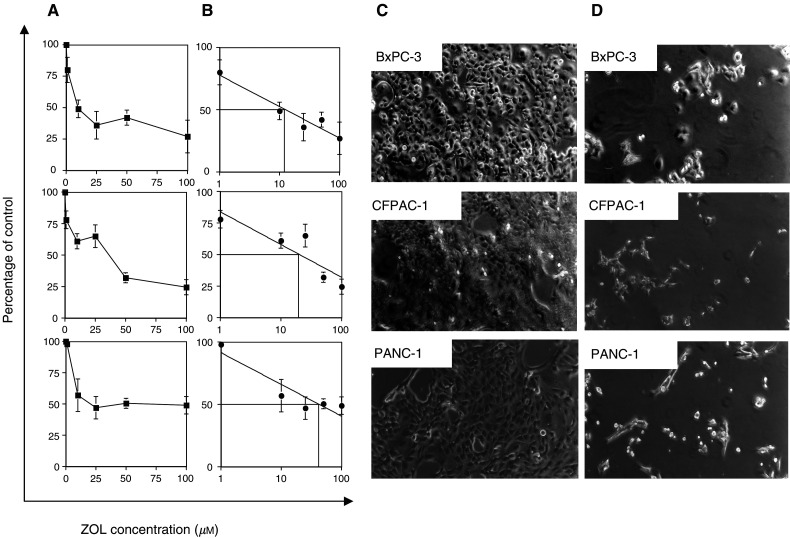
Zoledronic acid inhibits the growth of PC cells. (**A**) The cell lines BxPC-3, CFPAC-1 and PANC-1 (3 × 10^4^ ml^−1^) were seeded in 96-well plates and incubated for 24 h. After 24 h, medium was removed and replaced with medium containing increasing concentrations of ZOL (1–100 *μ*M). After 72 h of incubation, cell proliferation was determined by the MTT assay. A value of 100% was assigned to untreated control cultures, and the antiproliferative effects of ZOL were calculated as percentage of residual growth. Each point is the mean value of at least four replicate experiments ±s.e. (**B**) The IC_50_ drug concentration for each cell line was measured by a logarithmic interpolate curve on the percentage of residual growth at each concentration point (**C** and **D**) Morphological changes induced in cell cultures after 72 h exposure to ZOL. Analysis was performed by inverted phase-contrast microscopy. (**C**) Untreated cultures; (**D**) treated cultures with ZOL (50 *μ*M).

The effect of ZOL on BxPC-3, CFPAC and PANC-1 PC cells was investigated *in vitro* using the MTT assay. Treatment with ZOL (1–100 *μ*M) produced a dose-dependent reduction of cell growth after 72 h of treatment (Figure 1A

) and the IC_50_ was calculated in a range of 10–50 *μ*M (Figure 1B). Figure 1C and D show the morphological changes of cultured PC cells after 72 h exposure to ZOL (50 *μ*M). Untreated cells (Figure 1C) were flat and well spread, but exposure to the drug (Figure 1D) resulted in significant antiproliferative effects, retraction of cells from the substratum, rounding up and loss of contact between neighbouring cells. Altogether, these findings indicate that ZOL exerts growth inhibitory activity on PC cells.

### Zoledronic acid induces apoptotic death of PC cells

**Figure 2:**
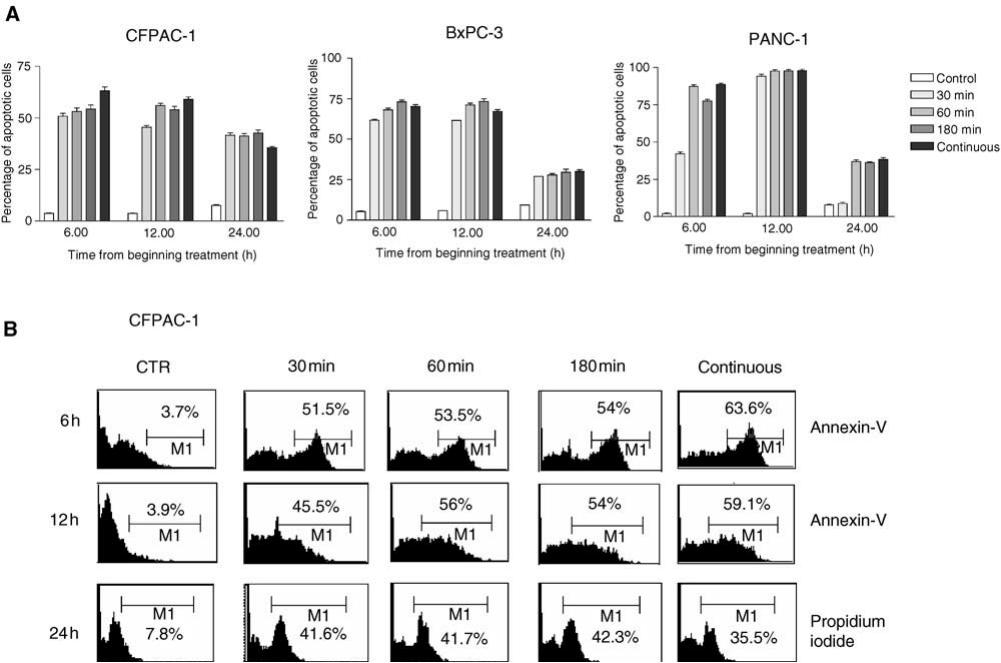
(A) Apoptotic effects induced by ZOL on CFPAC-1, PANC-1, BxPC-3 PC cancer cells. The analysis of apoptosis was performed after exposure to 50 *μ*M of ZOL by flow cytometric detection of Annexin-V immunostaining. Cells were pulse exposed for 30, 60 and 180 min or continuously exposed to the drug. The PI method was used when apoptosis was evaluated 24 h after beginning treatment. Data are expressed as the mean value of at least four replicate experiments ±s.e. (**B**) Flow cytometric profiles from a representative experiment performed on CFPAC-1 cells. The percentage of apoptotic stained cells (%) is indicated in each quadrant.

To clarify the mechanisms of ZOL-induced growth inhibition, we performed apoptotic assays on PC cells exposed to this compound. Activation of apoptosis was detected by Annexin-V staining, which is early expressed on the outer side of the cell membrane only when apoptosis is triggered and by PI which directly measures fragmented DNA. Figure 2

shows the apoptotic death of BxPC-3, CFPAC-1 and PANC-1 cells after 6, 12 and 24 h exposure to 50 *μ*M ZOL. Apoptotic cell death was detected in 35–90% of treated PC cells, suggesting a significant role of apoptotic death in the *in vitro* activity of ZOL. Notably, induction of the apoptosis was independent from the length of ZOL exposure. After 6–12 h from the beginning of ZOL exposure, Annexin-V staining was equally detected in cells treated by a 30–180 min pulse as compared with cells treated with continuous drug exposure, suggesting that induction of the apoptotic process is an early event in pancreatic cells exposed to ZOL. DNA fragmentation demonstrated by the PI experiment became evident after 24 h and again was independent from the length of exposure (pulse *vs* continuous).

### Caspase- and PARP-dependency of ZOL-induced apoptosis in PC cells

**Figure 3:**
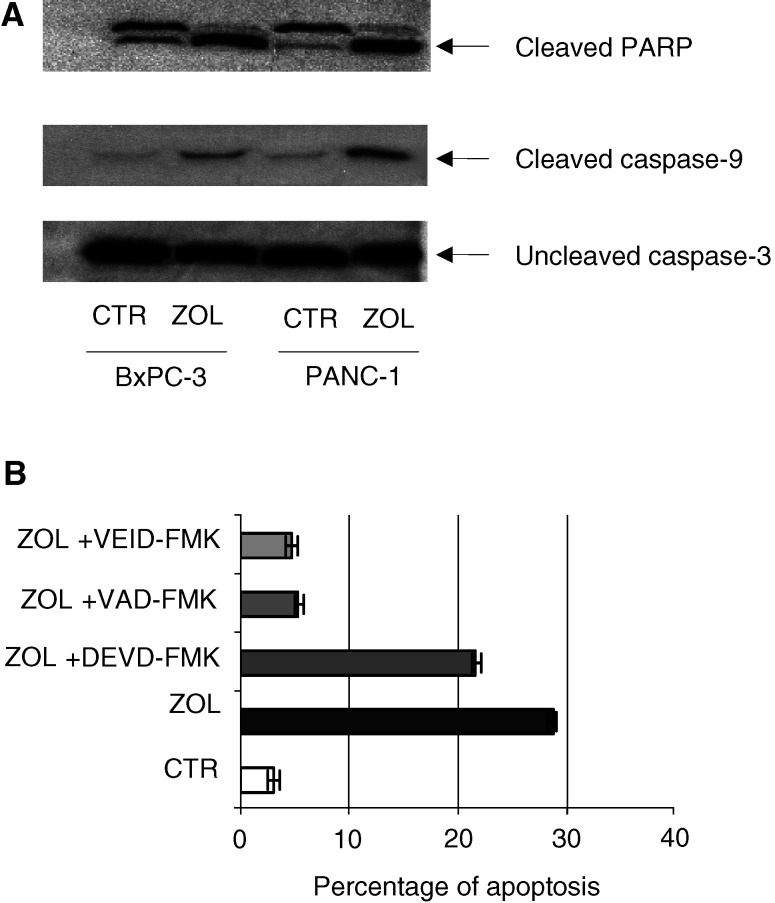
Role of caspase-9/-3 and PARP in apoptotic cell death induced by ZOL. (**A**) Detection of caspase-9/-3 and PARP in PC cells after ZOL treatment. Western blotting analysis was performed on protein extracts from solubilized whole cell pellets from BxPC-3 and PANC1 PC cell lines after 48 h exposure to ZOL (50 *μ*M) demonstrating abundant cleaved species of both caspase-9 and PARP in lysates for drug-exposed cells, while caspase-3 remained almost completely uncleaved. The experiments were performed at least three times and the results were always similar. (**B**) Apoptotic effects induced by ZOL in the presence of caspase inhibitors. Analysis was performed by flow cytometric immunodetection of Annexin-V staining on BxPC-3 cells after 48 h exposure to ZOL (50 *μ*M). VAD is a caspase-9 inhibitor, VEID is a specific caspase-6 inhibitor and DEVD is a caspase-3 inhibitor. Pretreatment with 15 *μ*M VAD and 15 *μ*M VEID significantly antagonised ZOL-induced apoptosis (*P*<0.005).

We investigated the molecular mechanisms of ZOL-induced apoptosis in these cells. We found that exposure of PC cells to ZOL increased PARP cleavage, a key enzyme in the apoptotic cascade, as demonstrated by Western blot analysis, which showed a higher expression of the 116 kDa proenzyme and the appearance of an 89 kDa cleavage product in treated cells. The maximal effect was detected after 72 h of ZOL exposure (Figure 3A

). We also found that caspase-9 was cleaved/activated after cell exposure to the drug, while no effect was detected on caspase-3. These data were confirmed by the use of specific caspase inhibitors. We demonstrated that the caspase-9 inhibitor Z-VAD and the specific caspase-6 inhibitor VEID almost completely prevented apoptotic death of PC cells, while the DEVD caspase-3 inhibitor only slightly affected apoptosis induced by ZOL, as detected by flow cytometric analysis of Annexin-V staining (Figure 3B). These findings suggest that apoptosis induced by ZOL on human PC cells is caspase-9-, caspase-6- and PARP-dependent, but caspase-3-independent.

### Zoledronic acid inhibits the p21^ras^/Raf1/MEK/ERK1-2 mitogenic and pKB/Akt antiapoptotic pathways in PC cells

**Figure 4:**
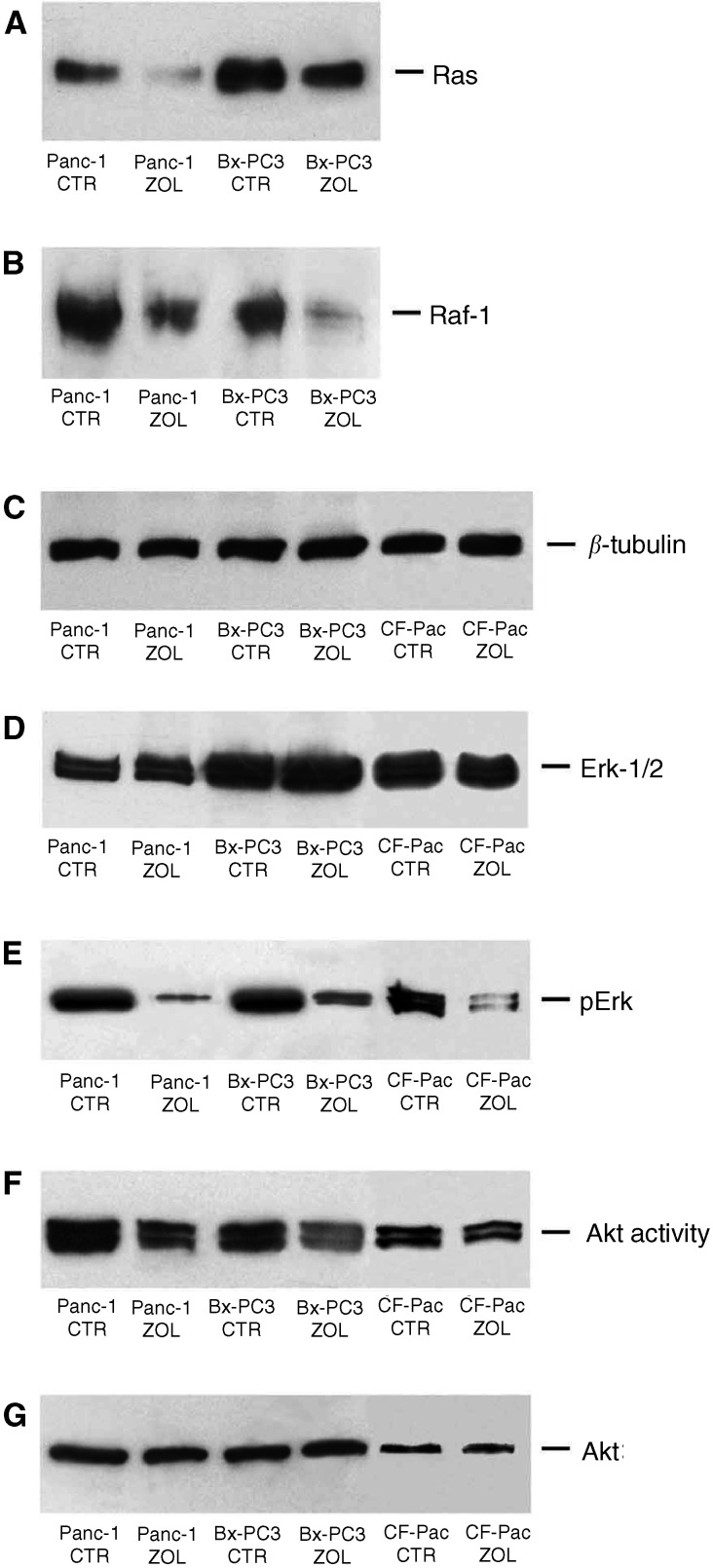
Effects of ZOL treatment on p21^ras^/Raf-1/MEK1/ERK signalling and PK-B/Akt pathways in PC cells. Western blotting analysis of p21^ras^ (**A**), raf-1 (**B**), Erk-1/2 (**D**) and pErk (**E**). The immunoblotting shows downregulation of p21^ras^ (**A**) and raf-1 (**B**) levels after 48 h of exposure to 50 *μ*M ZOL. While no significant effects were detected on Erk-1/2 expression after exposure of cells to the drug (**D**), the active phosphorylated Erk-1/2 (recognised by an anti-pERK Mab) levels were greatly reduced in ZOL-treated cells (**E**). (**F** and **G**) Akt expression and functional analysis in BxPC-3, PANC-1 and CFPAC-1 pancreatic adenocarcinoma cells. For detection of Akt activity, immunoprecipitation was performed from cell lysates with anti-Akt antibody, and an *in vitro* kinase assay was performed using GSK3*α*/*β* as a substrate, followed by Western blotting analysis with antiphospho-GSK3*α*/*β*. GSK3*α*/*β* phosphorylation by immunoprecipitated Akt was downregulated after 48 h treatment of PC cells with 50 *μ*M ZOL (**F**), whereas the Akt protein levels remained unmodified. The experiments were performed at least three times and the results were always similar. CTR: untreated cells; ZOL: ZOL-treated cells.

In order to investigate the molecular mechanism of ZOL-induced growth inhibitory activity, we analysed whether the drug would affect the function of the ras/raf1/MEK/ERK1-2 pathway. We observed a strong decrease of the intracellular content of p21^ras^ and of its substrate Raf-1 as evaluated by Western blotting (Figure 4A and B

). In fact, 48 h treatment with 50 *μ*M ZOL induced a two-to-three-fold reduction of the expression of the two signalling components (Figure 4A and B). Subsequently, we studied the effects of ZOL on the activity of ERK1-2, the terminal enzyme of the ras/raf-1 antiapoptotic and survival pathway. We found a strong decrease of the activity of the two enzymes, as demonstrated by the immunodetection of the phosphorylated forms of ERK1-2 (Figure 4E). On the other hand, no changes in the intracellular levels of ERK1-2 were found in the ZOL-treated PC cells (Figure 4D). These data suggest a significant inhibition of the signalling activity of this mitogenic and survival pathway. Another important and ras-dependent survival pathway of human cancer cells is mediated by the activation of the intracellular kinase pKB/Akt. Therefore, we also studied the effects of ZOL on Akt activity. We observed a significant impairment of pKB/Akt activity in cells exposed to ZOL as demonstrated by reduced phosphorylation of the GSK3*α*/*β* fusion protein induced by the immunoprecipitated Akt (Figure 4F). We conclude that ZOL interferes with signalling pathways, which mediate proliferative stimuli and protect tumour cells from triggering apoptosis.

### Zoledronic acid induces actin cytoskeletal reorganisation in PC cells

**Figure 5:**
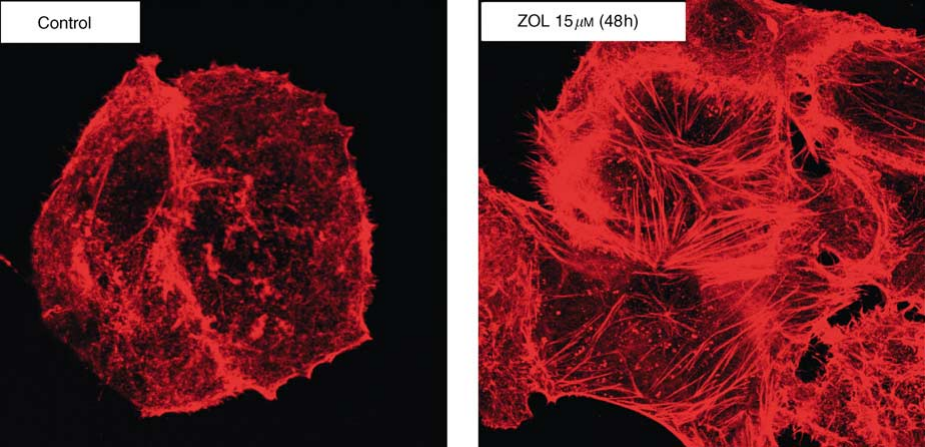
Analysis of cytoskeletal reorganisation by confocal microscopy on PANC-1 PC cells after 48 h exposure to ZOL (15 *μ*M). The figure shows actin architecture rearrangements in cortical rings. The cells were examined under a confocal microscope at a magnification of × 100. The experiments were performed at least three times and the results were always similar.

Finally, we studied whether the morphological changes induced in PC cells after exposure to ZOL occurred together with cytoskeletal reorganisation, which is considered to antagonise cell migration and invasion (Nobes and Hall, 1995). After 48 h exposure to ZOL (15 *μ*M), we observed reorganisation of the cytoskeleton and cortical actin polymerisation as judged by confocal microscopy detection of phalloidin-stained actin filaments. At these concentrations and exposure time, ZOL did not induce increased apoptotic death as compared with untreated cells. Figure 5

shows cytoskeletal rearrangements into cortical rings after the treatment. These findings suggest that the drug may trigger apoptotic death of PC cells, at least in part by interfering with cytoskeletal integrity.

## DISCUSSION

In this study, we have demonstrated that the most potent antiresorptive nitrogen-containing BP ZOL causes antiproliferative effects, perturbation of the p21^ras^/Raf1/MEK/ERK1-2 mitogenic pathway and pKB/Akt survival signalling, and also induces apoptotic death of human PC cells *in vitro*. We have also shown that the apoptotic events induced by the drug directly involve activation of caspase-9, caspase-6 and PARP. The present study provides the first evidence that a nitrogen-containing BP can directly interfere with intracellular mitogenic and survival pathways downstream to p21^ras^ and produce antitumour effects in cultured PC cells.

Bisphosphonates are an emerging class of drugs mostly used in the palliative care of cancer patients. These compounds are specific inhibitors of osteoclastic activity and may significantly reduce skeletal complications, thereby sustaining the quality of life of cancer patients. There is a growing interest in the possibility that BPs may also improve survival in these patients. Several properties of BPs have been recently highlighted. Experimental findings suggest that these drugs may directly act on cancer cells, either by inhibiting tumour cell invasion or adhesion to bone matrix or by inducing growth inhibitory and/or apoptotic cell death. This proapoptotic ability of BPs has been strongly correlated with the specific antiresorptive potency of each compound. Specifically, it has been demonstrated that ZOL has a direct effect on human multiple myeloma (Shipman *et al*, 1997), breast cancer (Senaratne *et al*, 2000), prostate cancer (Lee *et al*, 2001) and melanoma cells (Riebeling *et al*, 2002).

We found that ZOL induces significant antiproliferative effects in a range of 10–50 *μ*M. These data agree with most other *in vitro* studies showing maximal growth inhibitory effects of BPs at doses higher than what is persistently achievable in the serum of patients after treatment. In fact, it has been shown that infusion of Pamidronate, which is a less potent antiresorptive BP commonly used in palliative treatments, gives serum concentrations ∼10-fold lower (0.5–8 *μ*M) (Daley-Yates *et al*, 1991; Oiso *et al*, 1994; Berenson *et al*, 1997).

It is well known that BPs have a rapid clearance from the blood stream and therefore only effects induced by short-term exposure have a clinical relevance for extra bone antitumour activity. We have indeed that induction of apoptotic death in PC cells occurs after only 30 min pulse exposure and does not require continuous drug exposure. We think therefore that this latter finding increases the potential translational fall-out of this study. Moreover, we consider of interest that PC has been reported to metastasize to bone in approximately 15% of the cases. The low rate of bone involvment is not unexpected, because only with the recent improvements in diagnosis and treatment PC patients begin to survive long enough to reach the stage of disease most associated with distant skeletal lesions. Even if it has not been stated which bone sites may be specifically involved in metastatic spread, it has been suggested that PC metastasises to bone in a pattern that predominantly favours the pelvic girdle (Lyons *et al*, 2001). These lesions may heavily affect the quality of life of advanced cancer patients and overall survival. Therefore, our data strengthen the rationale for the use of these drugs in a palliative treatment of skeletal metastatic disease. It has however to be underlined that palliative effects on the metastasis at the bone site do not necessarily involve direct antitumour activity.

The molecular bases of the BP antitumour activity remain to be elucidated. However, one possible mechanism is based on the ability of BPs to inhibit some of the enzymes involved in the pathway for cholesterol synthesis. Relevant to our study, dysregulation of p21^ras^ activity appears to be a critical event for the onset of PC. We have shown that ZOL interferes with the p21^ras^/raf-1/MEK1/ERK and pKB/akt signalling cascades, as p21^ras^ and Raf-1 contents were clearly reduced in PC cells, and the active phosphorylated species of ERK1-2-, and pKB/akt-mediated phosphorylation of GSK*α*/*β* were strongly downregulated in cells exposed to the drug. These data appear to be of specific interest considering the widely described growth promoting and antiapoptotic activity of the two p21^ras^-dependent raf-1/MEK1/ERK and pKB/Akt pathways, and our study provides, at our knowledge, the first evidence that ZOL affects the intracellular signalling pathway downstream to p21^ras^. Moreover, it has been demonstrated that Akt directly inhibits caspase-9 activity by phosphorylation and, intriguingly, we demonstrated that in our experimental model ZOL-induced apoptosis is caspase-9-dependent (Fujita *et al*, 1999; Zhou *et al*, 2000). In fact, we demonstrated that, in parallel with the antiproliferative effects mediated by the drug, ZOL also induces apoptotic cell death in PC cells. Cell death by an apoptotic process is a well-described phenomenon that is associated with multiple molecular events. The caspase family of cysteine proteases are central participants in apoptotic cell death. Recent studies have highlighted the potential role of caspase-9 as a selective target for anticancer treatment. It is also believed that chemotherapeutic agent-induced apoptosis is predominantly accomplished by activation of the mitochondrial pathway (Wu and Ding, 2002). In fact, blockade of caspase-9 decreases chemotherapeutic agent-induced mitocondrial-dependent apoptosis. In this context our observations are in agreement with other studies which reported that ZOL-mediated apoptosis is associated with cytochrome *c* release and consequent caspase activation (Senaratne *et al*, 2002). Caspase-3 is considered to be involved in the execution phase of apoptosis, when proteolysis of intracellular substrates is a major event. It has been shown that BP-induced osteoclast apoptosis is dependent on caspase-3 activation since cell death is prevented by caspase-3 inhibitors, thereby suggesting the key role of these proteases in apoptotic cell death induced by these drugs (Benford *et al*, 2001). Caspase-3 has also been involved in apoptotic death of breast cancer cells exposed to ZOL (Senaratne *et al*, 2002). In our study however, we have been unable to find a specific role for caspase-3, which is not cleaved/activated, and apoptosis was only slightly antagonised by selective caspase-3 inhibition. Our observations are in accordance with others who found no correlation between the amount of processing of caspase-9 and effector caspases in human pancreatic carcinoma (Gerhard *et al*, 2002). We can therefore hypothesise that in ZOL-exposed PC cells, a caspase-9- and caspase-6-dependent and caspase-3-independent pathway is operative, while in other tumour cell systems, as well as in normal osteoclast cells, execution of apoptosis induced by ZOL may occur by a caspase-3-dependent mechanism. The possible identification of tissue-specific executioners of apoptosis might be ideal subjects of investigation, and may have the advantage of enhancing selectivity in therapeutical intervention.

Activation of caspases during cell death is commonly associated with characteristic intracellular changes of *in vitro* cultured cells, including loss of adhesion, actin-cytoskeletal structural reorganisation into cortical rings, cell rounding and contracting, and generation of apoptotic bodies. A role for caspase-mediated proteolysis of structural and adhesion proteins has been suggested in the morphological changes that characterise apoptotic cell death. After the initial phase of contraction and blebbing, caspase-mediated cleavage of actin monomers probably induces disassembly of actin filaments (Mashima *et al*, 1997; McCarthy *et al*, 1997). We found that after 48 h of low concentration ZOL treatment, significant actin-cytoskeletal reorganization occurs in PC cells as demonstrated by actin staining. These modifications indicate that ZOL induces actin rearrangements into cortical rings and that these events may drive the cells to the apoptotic process. This specific effect indicates the intriguing possibility of positive interactions of BPs with drugs that interfere with actin polymerisation and depolymerisation such as taxoid or vinca alkaloid agents. A synergistic effect of ZOL with paclitaxel has in fact been demonstrated in human breast cancer (Jagdev *et al*, 2001). Recent reports have also highlighted the role of signal transduction pathways controlled by the Rho family of small GTPases in regulating the architecture of the actin cytoskeleton and the morphological changes during apoptotic cell death (Coleman and Olson, 2002). An intriguing preliminary finding is that the small GTP-binding protein RAC, functionally related to cytoskeletal organisation, discloses enhanced (10-fold) expression in ZOL-exposed cancer cells as detected by Western blot analysis (data not shown). Based on these findings, further studies are presently in progress in order to understand the functional significance of these observations.

In conclusion, we have demonstrated that PC cells are highly sensitive to ZOL-induced growth perturbation and induction of apoptosis, which is caspase-9- caspase-6- and PARP-dependent. Moreover, our experimental findings suggest that inhibition of p21^ras^/Raf-1/MEK1/ERK signalling as well as PK-B/Akt inhibition might be relevant for the antitumour effects of ZOL. Although we have been capable of demonstrating that a short-term exposure is enough for activation of apoptosis, we think that a pharmacokinetic profile more relevant to the extra-bone antitumour effects of ZOL should be derived and might involve entrapping of BPs in lysosomes. An additional topic of current investigation is BP combination with cytotoxic drugs or selective signal transduction inhibitors.
